# Primary Tubercular Breast Abscess in an Indian Female: A Rare Case

**DOI:** 10.7759/cureus.41586

**Published:** 2023-07-09

**Authors:** Sankalp Yadav

**Affiliations:** 1 Medicine, Shri Madan Lal Khurana Chest Clinic, New Delhi, IND

**Keywords:** cbnaat/ xpert/ rif assay, fine needle aspiration cytology (fnac), mtb (mycobacterium tuberculosis), tuberculosis, breast

## Abstract

Tuberculosis is widespread in developing countries, which usually presents as pulmonary tuberculosis. However, cases of extrapulmonary tuberculosis at sites other than the lungs are also reported. The present case is a rare variety of extrapulmonary tuberculosis presenting as a primary tubercular breast abscess in an Indian female with no pulmonary involvement and no history of tuberculosis. A detailed clinical workup led to the final diagnosis, and she was put on anti-tuberculous treatment per the national guidelines.

## Introduction

Tuberculosis poses a significant threat to public health [1]. The disease is caused by an infection with *Mycobacterium tuberculosis *and primarily spreads through the inhalation of aerosols contaminated with the bacteria [2]. Tuberculosis can be categorized into two types: pulmonary tuberculosis, where the primary infection occurs in the lungs, and extrapulmonary tuberculosis, which refers to infections located in sites other than the lungs [3]. Extrapulmonary tuberculosis constitutes about 17.5% of total tuberculosis cases [4].

Tuberculosis of the breast is a very rare entity [5]. Baharoon et al. mentioned that Sir Astley Cooper, in the year 1829, reported this clinical condition for the first time and called it “bosom’s scrofulous swelling” [6]. It constitutes only 0.1% of all cases of extrapulmonary tuberculosis and is commonly seen in females of the Indian subcontinent and Africa [4,7]. This percentage corresponds to about three percent of all breast diseases requiring surgical interventions [8]. In India, its incidence varies from 3% to 4.5% [9].

The present case is a very rare variety of extrapulmonary tuberculosis presenting as a primary tubercular breast abscess in a 52-year-old Indian female with no pulmonary foci and no history of tuberculosis. A detailed clinical workup led to the final diagnosis, and she was put on anti-tuberculous treatment per the national guidelines.

## Case presentation

A 52-year-old non-diabetic Indian female belonging to a low-income family presented with her primary concerns of a lump in her right breast for one and a half months, along with associated pain in the same breast for one month. She was in good health until 1.5 months ago, when she noted a lump in her right breast about 4 × 3 cm in size. It was insidious in onset, non-progressive in size, and associated with pain for one month. The pain was mild, non-radiating, and not associated with any aggravating or relieving factors.

There was no history of any other lump in the same breast, axilla, or opposite breast, and there was no fever, cough, or weight loss. Further, there was no history of trauma, hormonal therapy, tuberculosis contact, breast biopsy, or any other major medical or surgical intervention in the past. Furthermore, there was no history of substance abuse or imprisonment.

Obstetric histories were gravida - 4, para - 4, living - 4, and abortion - 0. She had her first child at the age of 26. She breastfed each child for 1-1.5 years. Her age at menopause was 50. A general and systemic examination was unremarkable. Local examination of the right breast revealed an ill-defined, tender, irregular, firm to hard lump about 4 × 3 cm in size with retroareolar extensions. It was not fixed to the skin or underlying chest wall, and there was a retraction of the nipple. Moreover, there was no cervical, supraclavicular, or axillary lymphadenopathy, clubbing, jaundice, pretibial edema, pallor, or cyanosis.

**Figure 1:**
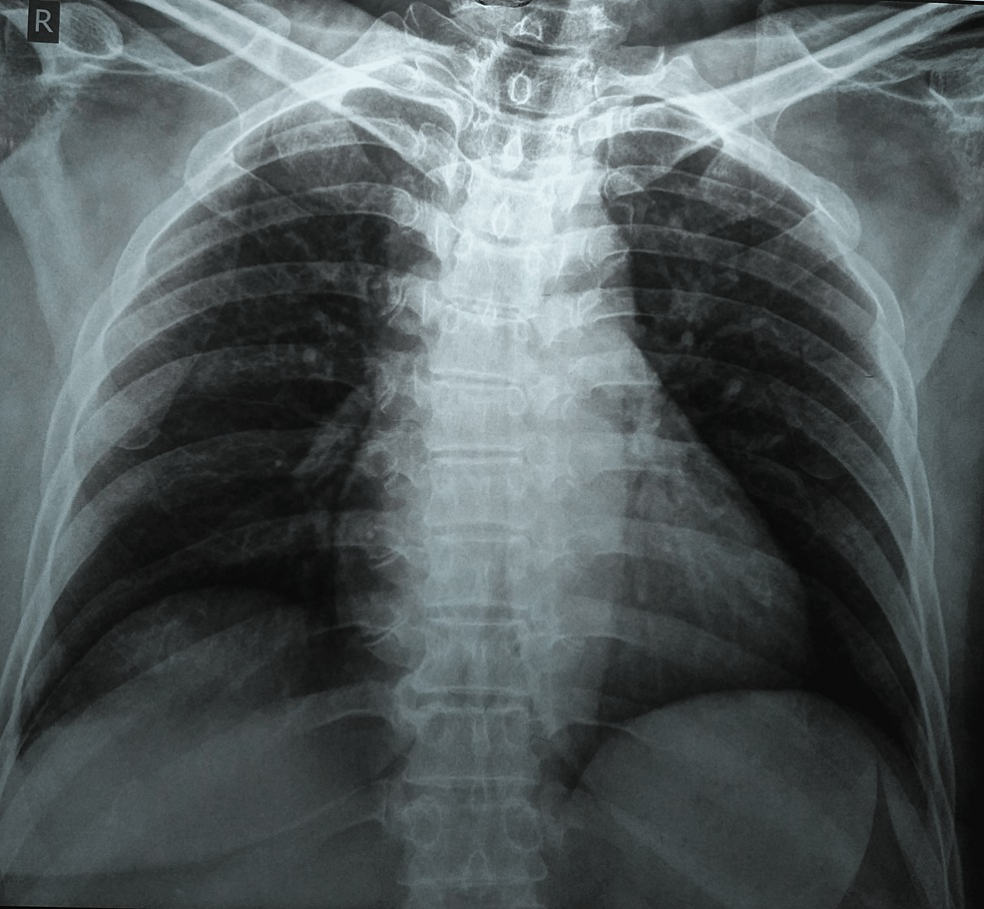
A chest radiograph (A-P view) within normal limits A-P: antero-posterior

A probable diagnosis of breast abscess was made with differentials for pyogenic breast abscess and breast carcinoma. And she was advised to have a chest radiograph, an ultrasound of the chest, and other routine blood investigations. Blood investigations were remarkable for a raised erythrocyte sedimentation rate of 57 mm in the first hour. The chest radiograph was within normal limits (Figure 1).

**Figure 2:**
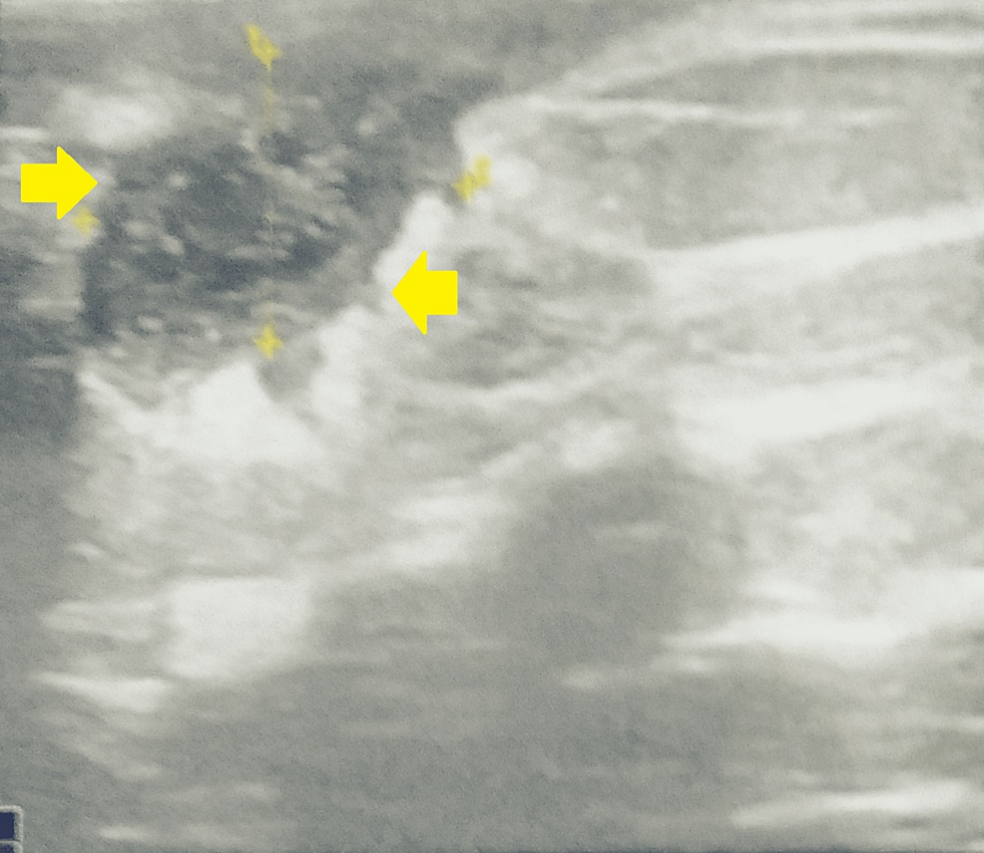
An ultrasound of the breast showing a heteroechoic collection in the right breast

An ultrasound of the breast revealed a heteroechoic collection of size 2 × 2.1 × 2.2 cm in the right breast with moving echoes predominantly in the retroareolar region (Figure 2).

**Figure 3:**
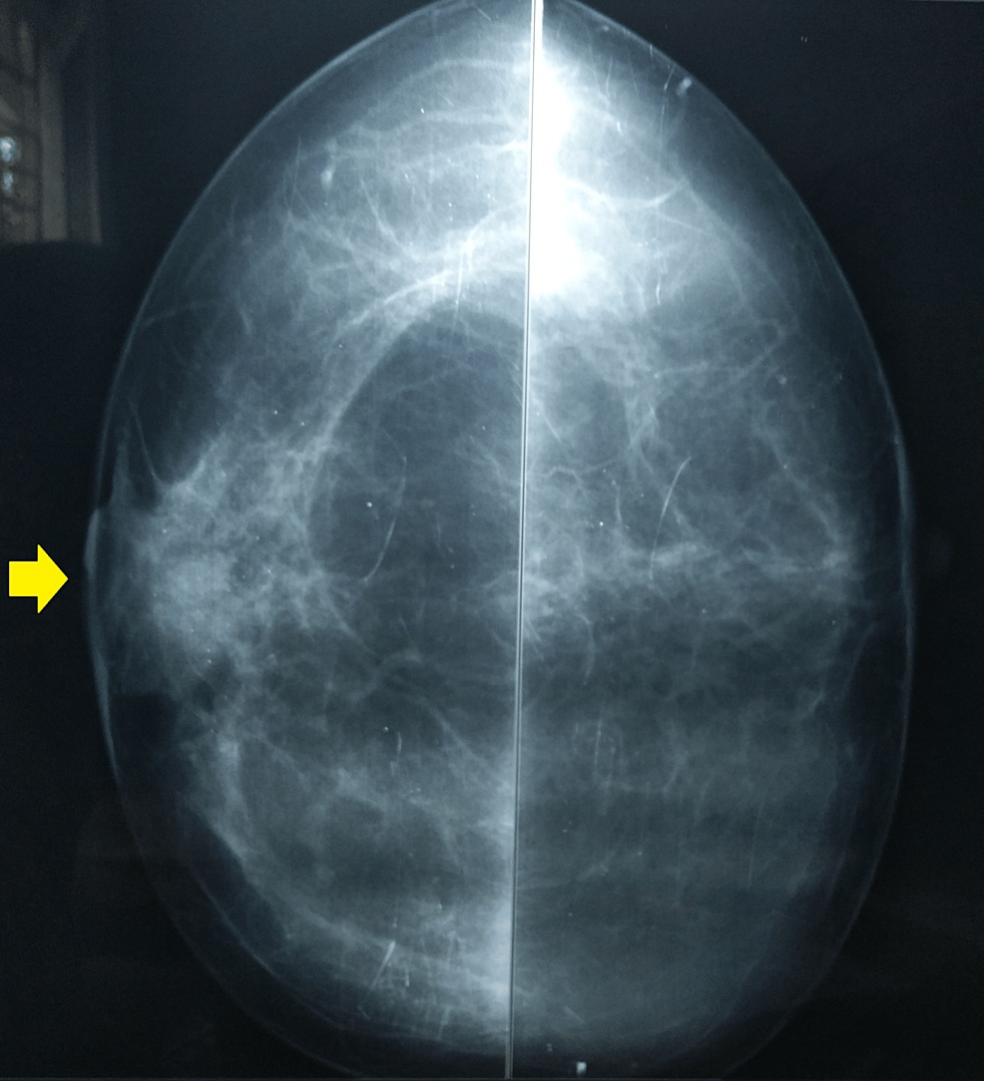
Mammogram bilateral CC-view showing well-defined opacity and retracted right nipple CC: craniocaudal

**Figure 4:**
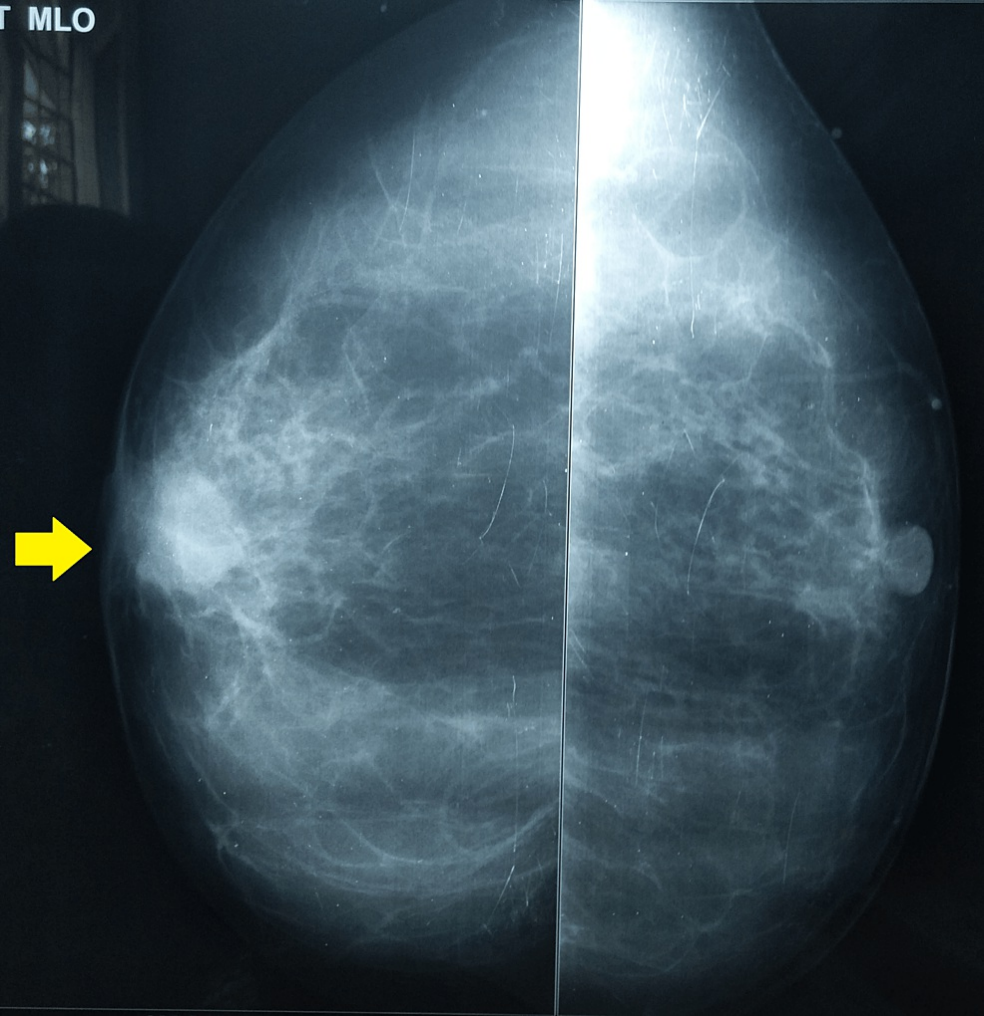
A breast mammography (MLO-view) showing opacity in the retroareolar region of the right breast MLO: mediolateral oblique

A breast mammography was suggestive of a 27 x 21 mm well-defined opacity in the retroareolar region of the right breast (Figures 3, 4).

A fine-needle aspiration cytology of the right breast led to the extraction of 30 cc of pus. The smear microscopy for acid-fast bacilli from the pus was positive for *Mycobacterium tuberculosis*. A cartridge-based nucleic acid amplification test of the pus was suggestive of medium detection of *Mycobacterium tuberculosis *with rifampicin sensitivity. One more sample was sent for culture and drug susceptibility testing, where *Mycobacterium tuberculosis *grew with no resistance to first-line anti-tubercular drugs. Additionally, a trucut biopsy revealed granulomas consisting of epithelioid cells, lymphocytes, and Langhans giant cells with no evidence of malignancy. An incision and drainage of the abscess and an excisional biopsy from the margin of the cavity were also done. Her tests for human immunodeficiency viruses I and II were non-reactive, and her Mantoux test was positive.

Finally, a diagnosis of primary tuberculosis of the right breast was made, and she was put on anti-tubercular drugs per her weight according to the national guidelines (isoniazid, rifampicin, pyrazinamide, and ethambutol for two months, followed by isoniazid, rifampicin, and pyrazinamide for four months). She responded well to her treatment with no adverse drug reactions and completed the full course with no residual lump. However, as she was transferred to her native village, a repeat ultrasound at the completion of her treatment was not accessible.

## Discussion

Tuberculosis is a significant contributor to morbidity and mortality in endemic countries [10]. The disease commonly manifests as pulmonary tuberculosis, but cases of extrapulmonary tuberculosis like tuberculosis of the breast are also available in the literature [8,9]. The disease has a female proclivity, but male breast tuberculosis is also reported [11]. Tuberculosis of the breast is usually seen in females 20-40 years of age, but no age group is spared [11,12]. It is classified as primary, i.e., present only in the breast and absent in other body parts, or secondary when foci other than the breast are also present in the body [12]. Secondary breast abscesses are relatively more common compared to the primary type [11].

Breast tuberculosis is commonly reported as a breast lump in the central or upper-outer quadrant; cases of multiple lumps are rare [12]. Primary breast tuberculosis, as seen in the present case, often results due to abrasions or through the openings present at the ductules of the nipple [11]. In the year 2009, Da Silva et al. reported abscesses and skin fistulations as the commonest lesions, the majority of which were noted in young women [8]. Khanna et al. (2002) and Asjad et al. (2019) reported that lactation could result in higher chances of tuberculosis of the breast in 33% and 69% of study subjects [11,13]. This could be attributed to trauma to the ductules associated with an increased blood supply to the breast tissue, which ultimately makes it more susceptible to inflammation [11,12].

Further, the diagnosis of tuberculosis of the breast is a challenging task [12]. The disease has non-specific clinical, radiological, and histological findings [14]. Often, the presentation overlaps with other similar conditions like pyogenic breast abscess or breast cancer [6]. The classical clinical presentations of tuberculosis, i.e., fever, cough, and weight loss are present in less than twenty percent of patients [12]. Misdiagnosis or delayed diagnosis is frequent because of the biopsy specimens, which are paucibacillary and usually have negative results on microscopy and culture [14].

The diagnosis could be achieved by investigations like the Ziehl-Neelsen stain, a cartridge-based nucleic acid amplification test, or culture of the aspirate [11]. Microbiological confirmation of *Mycobacterium tuberculosis* is usually reported in only 25% of cases [11]. Management is mostly conservative with anti-tubercular drugs with or without aspiration or surgical drainage of the pus, which could yield favorable outcomes [7].

A case similar to this was presented by Singal et al., where a 25-year-old female reported a right breast lump [14]. The present case shares similarities with theirs in site, primary foci in the breast, clinical features except for fever, raised erythrocyte sedimentation rate, a normal chest radiograph, and diagnosis with fine needle aspiration cytology [14]. However, the present case has a few unique features, like a diagnosis established by a cartridge-based nucleic acid amplification test and a culture of the pus with a positive Mantoux test.

Another case by Jadhav et al. shared findings with the present case in a primary right breast abscess, a normal chest radiograph, a raised erythrocyte sedimentation rate, ultrasound findings, and diagnosis with fine needle aspiration cytology and culture [9].

A very rare case of primary tubercular breast abscess in an Indian female is presented. The patient was diagnosed and managed with anti-tubercular drugs. There is a paucity of data about this condition, and this case highlights the importance of reporting such cases, especially in high-burden settings. Large-scale data could help in making new or modifying existing guidelines for management.

## Conclusions

Primary tuberculosis of the breast is a seldom reported condition. Often, these patients undergo empirical treatments before a final diagnosis is established. It requires a high index of suspicion backed by a strong diagnostic workup to achieve successful management. Primary care physicians should be trained in this clinical condition for timely treatment initiation.
